# Outsmarting cancer: the power of hybrid genomic/proteomic biomarkers to predict drug response

**DOI:** 10.1186/bcr3635

**Published:** 2014-03-31

**Authors:** Brent N Rexer, Carlos L Arteaga

**Affiliations:** 1 Department of Medicine, Vanderbilt-Ingram Cancer Center, Vanderbilt University, 2220 Pierce Avenue, 777 PRB, Nashville, TN 37232, USA; 2 Department of Cancer Biology, Vanderbilt-Ingram Cancer Center, Vanderbilt University, 2220 Pierce Avenue, 777 PRB, Nashville, TN 37232, USA; 3 Department of Breast Cancer Research Program, Vanderbilt-Ingram Cancer Center, Vanderbilt University, 2220 Pierce Avenue, 777 PRB, Nashville, TN 37232, USA

## Abstract

A recent study by Niepel and colleagues describes a novel approach to predicting response to targeted anti-cancer therapies. The authors used biochemical profiling of signaling activity in basal and ligand-stimulated states for a panel of receptor and intracellular kinases to develop predictive models of drug sensitivity. In some cases, the response to ligand stimulation predicted drug response better than did target abundance or genomic alterations in the targeted pathway. Furthermore, combining biochemical profiles with genomic information was better at predicting drug response. This work suggests that incorporating biochemical signaling profiles with genomic alterations should provide powerful predictors of response to molecularly targeted therapies.

## 

Significant improvements in cancer therapy have come from identifying and targeting oncogenic drivers in specific tumor types. In breast cancer, targeting estrogen receptor-positive tumors with anti-estrogens and human epidermal growth factor receptor (HER)2-positive cancers with HER2 inhibitors represent significant advances. Recent efforts to find additional oncogenic drivers on a larger scale have focused primarily on high-throughput DNA sequencing. These studies have identified somatic alterations (mutations, amplifications, deletions, and so on) in a number of genes encoding signaling proteins [1,2]. There is considerable interest in using these mutations to identify patients for treatment with inhibitors of the altered signaling molecules. Questions remain as to whether those genetic lesions confer aberrant activity and, thus, tumor dependence on the altered signaling pathway and, if so, whether they predict a therapeutic vulnerability exploitable with a targeted inhibitor [3].

Moreover, although many drugs targeting signaling pathways are available, clinical responses are generally seen in only a minority of tumors with alterations in the targeted aberrant pathway. Many studies have focused on understanding what determines and how to predict the response to a molecularly targeted drug. Recent efforts such as the *Cancer Cell Line Encyclopedia* have leveraged large cell line panels to identify genomic predictors of response to treatment [4]. Despite these efforts, critical questions remain as to what signaling pathways are driving breast cancer subtypes, or mediating resistance to initially effective targeted therapies, and how to select the appropriate inhibitors for the tumor of a given patient.

Recent work by Niepel and colleagues [5] takes a novel approach, focusing on the biochemistry of signaling pathways rather than only genomic alterations. These authors investigated whether profiles of signal transduction networks before and after stimulation with ligands can predict sensitivity to drugs targeting those signaling pathways. They first measured the abundance and phosphorylation state of receptor tyrosine kinases (RTKs) and intracellular signal transducers under basal and ligand-stimulated conditions. They demonstrated that receptor abundance and activation often vary in a graded fashion within and across breast cancer subtypes. They then used the signaling profiles to cluster the cell lines into groups that partially, but not completely, overlap with clinical subtypes. In some cases, RTKs such as hepatocyte growth factor receptor and fibroblast growth factor receptor differentiated between the clinical subtypes; in others, outliers within the clinical subtype could be identified by their signaling profile. Finally, they used the signaling profiles to build models that successfully predicted response to 23 of the 43 targeted therapies using the half-maximal growth inhibitory concentration for drugs in the Integrative Cancer Biology Program cell line panel (ICBP43) [6].

Using phosphoinositide 3-kinase (PI3K)-Akt inhibitors as an example, Niepel and colleagues showed how their models can be used to identify which signaling parameters are most important to determining response. For PI3K inhibitors, signaling through Erk in response to the HER3 ligand heregulin and HER3 abundance, but not phosphoinositide 3-kinase catalytic subunit alpha (*PIK3CA*) mutations, were key predictors of response. They also showed that HER2 and HER3 receptor abundance together, perhaps acting as a surrogate for heregulin-stimulated Erk, predicted sensitivity to PI3K pathway inhibitors. Combining genomic information with the signaling profiles significantly improved the predictive power of the models. Using signaling profiles alone, predictive models could be developed for 6 of 11 PI3K pathway inhibitors, but these profiles together with *PIK3CA* or *PTEN* mutational status predicted the activity of 10 of 11 inhibitors.

Despite its novelty, this approach has limitations for its applicability to clinical investigation. It uses cell lines in culture, depends upon the ability to measure multiple parameters after perturbations in a controlled system, and focuses mainly on ErbB-PI3K pathway signaling. However, the findings are significant for several reasons. First, they serve as a proof of principle that biochemical profiling on a relatively large scale can predict drug responses. Importantly, modeling of these profiles can identify a few nodes that reflect the activity of larger signaling networks. Second, this work augments the predictive power of genomic and expression profiling and shows how combining biochemical phenotypic data with genomic data can improve the predictive power of both. Third, the incorporation of ligand-stimulated signaling to the modeling adds at least a rudimentary approximation of what is likely occurring in patient tumors exposed to multiple ligands in the tumor microenvironment. Finally, biochemical profiling of patient tumors is currently not feasible on a large scale, but could potentially be performed for those few species predicted to reflect the larger signaling program. A number of issues complicate the measurement of dynamic signaling events in primary tumors, particularly given that ligands cannot be administered to patients. However, presurgical or neoadjuvant studies might be used for measuring not activation but interruption of signaling after treatment with a particular inhibitor, as has been shown for epidermal growth factor receptor inhibitors and anti-estrogens, for example [7-9].

Another promising approach is to use signaling network profiles, such as those described in this work, to refine therapeutic genomic predictors. Studies such as The Cancer Genome Atlas have given us important information on genomic alterations in signaling molecules and pathways, but investigation into how these genotypes affect the signaling phenotypes of breast cancer cells remains limited. The paradigm in this work exemplified by combining *PIK3CA* mutations with response to PI3K inhibitors could be extended to other breast tumor subtypes and driver oncogenes. This work also demonstrates that ‘hybrid’ biomarkers combining specific genomic alterations with markers of signaling pathway activation are likely to be required to optimally predict response to targeted therapies.

### Abbreviations

HER: Human epidermal growth factor receptor; PI3K: Phosphoinositide 3-kinase; PIK3CA: Phosphoinositide 3-kinase catalytic subunit alpha; RTK: Receptor tyrosine kinase.

### Competing interests

The authors declare that they have no competing interests.
